# Potential Health Risk of Endocrine Disruptors in Construction Sector and Plastics Industry: A New Paradigm in Occupational Health

**DOI:** 10.3390/ijerph15061229

**Published:** 2018-06-11

**Authors:** Aleksandra Fucic, Karen S. Galea, Radu Corneliu Duca, Mounia El Yamani, Nadine Frery, Lode Godderis, Thórhallur Ingi Halldorsson, Ivo Iavicoli, Sophie Ndaw, Edna Ribeiro, Susana Viegas, Hanns Moshammer

**Affiliations:** 1 Institute for Medical Research and Occupational Health, 10 000 Zagreb, Croatia; afucic@imi.hr; 2 Institute of Occupational Medicine (IOM), Centre for Human Exposure Science (CHES), Edinburgh EH14 4AP, UK; karen.galea@iom-world.org; 3 Centre for Environment and Health, University of Leuven, 3000 Leuven, Belgium; radu.duca@kuleuven.be (R.C.D.); lode.godderis@kuleuven.be (L.G.); 4 Santé Publique France, French National Public Health Agency, 94 415 Saint-Maurice, France; Mounia.ELYAMANI@santepubliquefrance.fr (M.E.Y.); nadine.frery@santepubliquefrance.fr (N.F.); 5 IDEWE, Knowledge, Information and Research Center, 3001 Heverlee, Belgium; 6 Unit for Nutrition Research, The National University Hospital of Iceland, 101 Reykjavik, Iceland; tih@hi.is; 7 Faculty of Food Science and Nutrition, School of Health Sciences, University of Iceland, 101 Reykjavik, Iceland; 8 Department of Public Health, University of Naples Federico II, 80131 Naples, Italy; ivo.iavicoli@unina.it; 9 Institut National de Recherche et de Sécurité, Vandoeuvre CEDEX, 54500 Vandœuvre-lès-Nancy, France; sophie.ndaw@inrs.fr; 10 H&TRC—Health & Technology Research Center, ESTeSL—Escola Superior de Tecnologia da Saúde, Instituto Politécnico de Lisboa, 1990-096 Lisboa, Portugal; edna.ribeiro@estesl.ipl.pt (E.R.); susana.viegas@estesl.ipl.pt (S.V.); 11 Landscape, Environment, Agriculture and Food, Instituto Superior de Agronomia, Universidade de Lisboa, Tapada da Ajuda, 1349-017 Lisboa, Portugal; 12 Centro de Investigação e Estudos em Saúde Pública, Escola Nacional de Saúde Pública, Universidade Nova de Lisboa, 1600-560 Lisboa, Portugal; 13 Center for Public Health, Medical University of Vienna, 1090 Vienna, Austria

**Keywords:** endocrine disruption, construction sector, plastics industry, fertility, biomarkers

## Abstract

Endocrine disruptors (EDs) belong to large and diverse groups of agents that may cause multiple biological effects associated with, for example, hormone imbalance and infertility, chronic diseases such as diabetes, genome damage and cancer. The health risks related with the exposure to EDs are typically underestimated, less well characterized, and not regulated to the same extent as, for example, carcinogens. The increased production and utilization of identified or suspected EDs in many different technological processes raises new challenges with respect to occupational exposure settings and associated health risks. Due to the specific profile of health risk, occupational exposure to EDs demands a new paradigm in health risk assessment, redefinition of exposure assessment, new effects biomarkers for occupational health surveillance and definition of limit values. The construction and plastics industries are among the strongest economic sectors, employing millions of workers globally. They also use large quantities of chemicals that are known or suspected EDs. Focusing on these two industries, this short communication discusses: (a) why occupational exposure to EDs needs a more specific approach to occupational health risk assessments, (b) identifies the current knowledge gaps, and (c) identifies and gives a rationale for a future occupational health paradigm, which will include ED biomarkers as a relevant parameter in occupational health risk assessment, surveillance and exposure prevention.

## 1. Introduction

Endocrine disruptors (EDs) belong to a wide range of different substances, which interfere with mammalian hormone biosynthesis, transport, metabolism and receptors, affecting homeostasis, development, and aging, reduce fertility in both sexes and are linked with the etiology of both malignant and non-malignant diseases [1,2,3,4,5,6]. The biological effects of EDs are complex and do not only depend on dose, but also on the timing of exposure and the sex of the exposed subject [7]. The complex interplay between timing and dose can lead to a broad array of different effects [8]. Effect monitoring therefore requires a battery of specific biomarkers [9]. Moreover, adverse effects linked to ED cannot be forewarned by the classic dose-exposure-response relationship due to their nonlinear dose/effect relationship [10]. Thus, EDs represent a demanding and challenging issue within both the scientific and regulatory context [9,11].

Among the many known effects of EDs, those on male and female reproduction are of especially high concern [5,8]. Knowledge about health risks of EDs and about relevance of exposure reduction is still poor among pregnant women [12]. Similarly, knowledge among workers of both sexes on possible effects of ED on their fertility is certainly not better. While for some effects, late pregnancy might be the most critical time period [13], other effects are of higher concern if exposure occurs in early pregnancy [14,15] or even before the woman is aware of her pregnancy. These early developmental exposures could have long lasting effects in the offspring [16].

Additionally, in construction workers, a significant increase of esophageal, colorectal and gastric cancer was reported [17]. Increased cancer risk (in particular for colorectal, pleural and bladder cancer) was also found in residents living in the vicinity of cement, lime and plaster plants [18]. Testicular cancer was significantly increased in both construction and plastic industry workers [19]. All these cancer types are associated with disturbances of estrogen/testosterone ratio or of their receptors [20,21,22,23,24,25].

## 2. The Need for a New Paradigm in Occupational Health

In some occupational settings, exposures to EDs are likely to be frequent but also underreported, insufficiently monitored and lacking clear documentation of associations with health effects. Job–Exposure Matrices (JEM) have been developed for that purpose (e.g., [26,27]), but are far from sufficient. To address these deficits, a new paradigm of occupational health assessment, preventive measures and improvements to their regulation is crucial.

European regulations on phthalates, bisphenol and some other EDs have been introduced to reduce health risks of the general population. EU Regulation 2016/2235 added Bisphenol A (BPA) to the REACH Annex XVII Restricted Substances List. This Regulation also restricts the use of some phthalates in polyvinyl chloride (PVC) production and other plasticized materials in all toys and childcare articles, indirectly reducing occupational exposure by the reduced production [28]. Occupational exposure to BPA in inhalable dust is regulated in some countries but not all, although in 2014 the Scientific Committee on Occupational Exposure Limits recommended an 8-h time-weighted average (TWA) of 2 mg/m^3^ (as inhalable dust) and a biological limit value (BLV) of 7 µg/L (urinary total bisphenol-A) [29].

In this short communication, the construction and plastics industries were selected as examples of two sectors, in which many workers across Europe are exposed to EDs. These include phthalates, bisphenols, aniline, cadmium and chromium [30,31,32,33,34,35,36,37,38].

## 3. The Construction and Plastic Industries

With an annual turnover (total sales) above €1.2 billion in 2016, the construction sector (including its extended value chain) is the largest single activity and biggest industrial employer (42.9 million direct and indirect jobs; 28.9% of industrial employment) in Europe (European Construction Industry Federation, Press Release, “Construction industry continues its slow recovery process”, 9 June 2017). In 2016, the construction sector amounted to 8.6% of the EU’s total Gross Domestic Product. The European plastic industry turnover was €350 billion in 2016, with over 1.5 million workers [39]. Global plastics production is projected to reach up to 1.2 billion tons annually by 2050 [40]. In Europe, 20% of plastics production is used in the construction sector [41] as a response to near zero energy building and zero waste policies in Europe [42,43,44]. In order to achieve these demands, the construction sector has introduced different classes of plastics, waste and nanomaterials. These new materials retain building structural quality and help seal toxic compounds from waste used as construction material [45,46]. However, there is no knowledge of the possible interaction of plastic materials with building materials, which may cause biological effects (synergistic and/or additive effects), and consequently no occupational safety protocols for work with such complex mixtures containing ED.

The increasing need for energy-efficient, sustainable buildings has also resulted in the introduction of new construction and finishing materials made or containing plastic components for water and thermal insulation, flooring, glazing, windows, doors, roofs, paints etc. [47,48]. Plastic monomers and polymers are also used for the production of different concrete types. Additionally, the construction sector produces a huge amount of demolition waste, which may contain heavy metals and other EDs [49]. The EU Construction & Demolition Waste Management Protocol of 2016 plans special management measures to be implemented for asbestos. However, agents that may have ED properties are not included [50].

## 4. Discussion on EDs in Construction and Plastic Industry Technologies

### 4.1. Plastisizers

There are very few papers on the association of phthalate metabolites levels in plastics industry male workers with the disturbance of estrogen, testosterone levels, sperm motility and testicular cancer [51,52,53]. An increased risk of infertility in women exposed in the plastic industry has been reported [54]. Occupational studies performed in BPA manufacturers and epoxy resin industries have correlated potential health effects with detected BPA levels in human biological samples. These studies, focused on male vulnerability, report sexual dysfunction, endocrine disruption and epigenetic alterations [55]. Epidemiological evidence has shown significant effects on the offspring of parents exposed to BPA during pregnancy [56,57]. Although BPA is partly substituted with bisphenol F (BPF), it has recently been shown in in vitro and in vivo studies that BPF is more estrogenic than BPA [58,59,60]. Similarly, bisphenol S and bisphenol B are hormonally active in the same way as BPA or even to a greater extent [60,61]. No such studies were evident in the public domain for the construction sector, although exposure to EDs is likely to occur in those employed in finishing and recovery activities, based on emissions data from materials such as vinyl wall and floor coverings [62,63]. Additionally, animal models suggested that pregnancy represents a highly vulnerable period for women with possible risk of diabetes type 2 [64]. Although this warrants further investigations the results might have significant impact on occupational protection of pregnant women.

During the last decade, a new generation of phthalates were introduced that were believed to cause less effects on the human endocrine system. For example, di-isononyl phthalate (DINP) is used as a plasticizer in PVC roof sheets and along with di-isodecyl phthalate (DIDP replacing di-2-ethylhexylphthalate (DEHP)); it is also used in the flooring industry [65]. There is a significant lack of data on endocrine potency of these replacement phthalates. This highlights a need for further investigation especially as in silico results show they interfere with human sex hormone-binding globulin [66] and increase insulin resistance [67]. In 2002, the plasticizer 1,2-cyclohexane dicarboxylic acid diisononyl ester (DINCH) was introduced in the European market as a substitute for DEHP. However, animal models recently show that DINCH is also an ED, which raises the need for further investigation in the human population [68].

### 4.2. Heavy Metals and Metalloestrogens

Heavy metals and metalloestrogens such as cadmium, cobalt, copper, nickel, hexavalent chromium (Cr(VI)), lead, and mercury are EDs that couple with estrogen receptors and are significantly present both in construction materials and the plastics industry [16,69,70]. Application of fly ash has been reported to significantly increase the risk of exposure to heavy metals in the construction sector [71]. Additionally, despite regulations concerning the application of paint and materials containing heavy metals, they are still in use [72].

New materials to improve the energy efficacy of façades have been introduced, such as cadmium telluride modules [73]. Cadmium carcinogenicity mechanisms include estrogen as one of the signaling pathways [74,75].

Chromium is present in the cement and exposure to cement has been found to be correlated with a higher risk of oligospermia [76] and decreased testosterone levels in exposed men [77]. According to recent data, Cr(VI) causes sex specific lung cancer risk [78,79]. Paternal occupational exposure to Cr(VI) is associated with an increased risk of spontaneous abortion in their partners [80]. Although heavy metals in waste used as building materials are sealed, their immobilization is not always equally achieved [81], representing a health risk to workers and future residents. Since Directive 2003/53/EC came into effect [82], all EU Member States have been obliged to reduce the chromate content in cement and cement-containing preparations. Additionally, Regulation No. 1907/2006 (EC, 2006) defines that cement and cement-containing preparations may not be used or placed on the market if they contain, when hydrated, more than 2 ppm of soluble Cr(VI) of the total dry weight of the cement. The implementation of this Regulation has already showed results in a significant reduction of occupational allergic contact dermatitis in France and the UK [83]. However, in other parts of the World, such as Australia or India, exposure to chromium in cement still remains a problem [84,85,86].

Lead is a well-known ED [69,87,88] and has been historically used in many different construction materials and paints. As of early 2016, 36% of countries worldwide have established legally binding limits on lead in paint. Australia, Macedonia, Montenegro, New Zealand, Philippines, Serbia, and Thailand have established a complete ban on lead additives [89]. However, the presence of lead in construction waste can be significant and the risk of exposure is high, especially due to extensive international waste market.

As the only renewable building material, wooden constructions are recommended [90,91,92]. Despite the fact that long-term wood preservers such as chromated copper arsenate are banned [93], they still represents a problem in the demolition and waste sectors which need detoxification and special workers protection.

### 4.3. Other Agents

Examples of other hazardous substances that are reported to have ED properties are also evident in the published literature. While the following is not a comprehensive list, some examples are provided below.

Current anti-fungal wood preserving technologies including the application of azole compounds have been reported as being aromatase inhibitors and antiandrogens [94] and fetotoxic in animal models [95]. Cypermethrin, a synthetic pyrethroid, used as an insecticide has anti-estrogenic activity [96].

Nonylphenol, used as additive in cement and mortar production, binds to estrogen receptors [97,98].

Aniline is used in shellacs for wood coloring; thus, construction painters and finishing workers are potentially exposed. Aniline is metabolized in paracetamol, which is an antiandrogen substance [36,37], causing hormonal disturbances [99].

Nanoparticles, such as titanium dioxide, silicon dioxide and aluminum, which are commonly used as components of construction materials, have also been shown to interact with endocrine system [33].

## 5. Conclusions

The effects of carcinogens are generally well known, and they are regulated more strictly than those for reprotoxic chemicals. Given that xenobiotics may act as EDs at lower doses than they act as carcinogens, pertinent permissible levels to protect worker should be put in place.

Current health surveillance of workers in the construction sector and plastic industry gives very limited insight into the health risks associated with exposure to EDs [100], although the need for the introduction of biomarkers of exposure in health risk assessments has already been recognized [101]. It is considered that the introduction of specific biomarkers of occupational exposure to ED should be included in occupational health surveillance, similar to the collection of anamnestic data on fertility problems, miscarriages and early menopause. Therefore, besides measuring EDs or their metabolites in biological samples, the inclusion of estradiol and testosterone levels which are possible to measure simultaneously with blood screening would already give insight in possible hormonal disturbances without significant increase in biomonitoring costs.

In the plastics industry and construction sector, workers are exposed to complex mixtures of xenobiotics, the effects of which are combined with the lifestyle, diagnostics (radiation) and diet of each person (as is the case for workers in the majority of other industries). Therefore, one of major priorities moving forward is the development of a personal biomonitoring tracking system linked to personal medical records that enables more accurate preventive measures and diagnostics.

**Figure 1:**
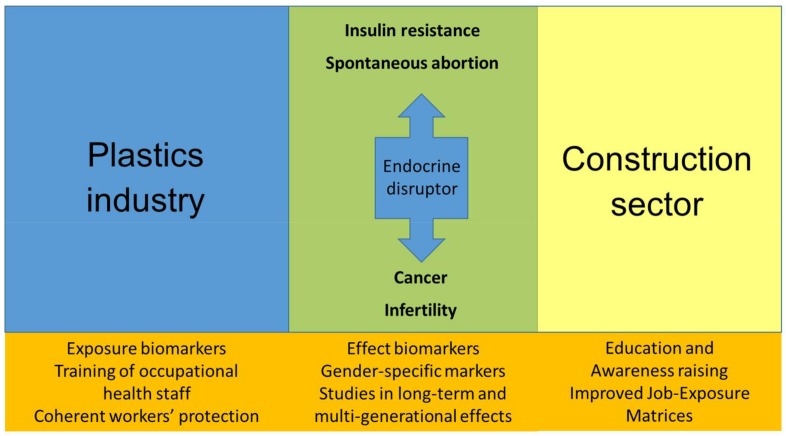
Schematic presentation of the topics and avenues for further research.

In order to achieve this challenging aim, summarized in Figure 1, the authors suggest the following:

(a) Fostering of collaborative studies with a view to collecting and sharing data of biomarkers of occupational exposure to EDs initially in construction sector and plastics industry
(b) Promotion of studies to get further insight in estrogen and testosterone levels and fertility problems on workers occupationally exposed to EDs
(c) Inclusion of sex-specific analyses of occupational health risk
(d) Education of occupational physicians and hygienists, employers and employees and other key stakeholders concerning the EDs co-exposures.
